# Stimulated saliva composition in patients with cancer of the head and neck region

**DOI:** 10.1186/s12903-021-01872-x

**Published:** 2021-10-09

**Authors:** Ulrica Almhöjd, Hulya Cevik-Aras, Niclas Karlsson, Jin Chuncheng, Annica Almståhl

**Affiliations:** 1 Department of Cariology, Institute of Odontology, Sahlgrenska Academy, University of Gothenburg, Gothenburg, Sweden; 2 Department of Oral Pathology and Medicine, Institute of Odontology, Sahlgrenska Academy, University of Gothenburg, Gothenburg, Sweden; 3 NÄL Hospital, Trollhättan, Sweden; 4 BioMS, Institute of Odontology, Sahlgrenska Academy, University of Gothenburg, Gothenburg, Sweden; 5 Department of Oral Microbiology and Immunology, Institute of Odontology, Sahlgrenska Academy, University of Gothenburg, Gothenburg, Sweden

**Keywords:** Head and neck cancer, Stimulated whole saliva, Salivary secretion rate, Total protein, Immunoglobulin A, MUC5B, MUC7, *O*-Glycosylation

## Abstract

**Background:**

To analyse over time changes in stimulated whole saliva regarding total protein, Immunoglobulin A (IgA), and mucin type O-glycans (mostly MUC5B and MUC7) in head and neck cancer patients.

**Methods:**

29 dentate patients (20 men and 9 women, 59 ± 8 years) treated with curative radiation therapy and chemotherapy for cancer of the head and neck region were included. The stimulated whole salivary secretion rate was determined and saliva collected at four time-points: at pretreatment, and at 6 months, 1 and 2 years post treatment. The total protein concentration was determined spectrophotometrically by using Bicinchoninic Acid assay and Immunoglobulin A (IgA) by using ELISA technique. Glycosylation pattern of salivary mucins was determined in samples collected pre- and post treatment by using LC/MS electrospray and mucin content quantified using SDS-AgPAGE gels and PAS staining.

**Results:**

Compared with pretreatment, the total protein concentration was increased already at 6 months post treatment (*p* < 0.01), and continued to increase up to 2 years post treatment (*p* < 0.001). During that period no significant changes in IgA concentration was detected. At pretreatment, the output/min of both total protein and IgA was significantly higher than at all time-points post treatment. Saliva from the cancer patients showed a low abundance/no detectable MUC7, while the MUC5B level remained, compared to saliva from a healthy control. The glycomic analysis showed that the percentage of core 2 *O*-glycans was increased as core 1, 3 and 4 *O*-glycans were decreased. The level of sialylation was higher at 6 months post treatment, while sulfation was lower.

**Conclusion:**

A decreased output per minute of proteins at decreased salivary secretion rate, as well as reduced sulfation of MUC5B at 6 months post treatment tended to correlate with the patients’ experience of sticky saliva and oral dryness. At 2 years post treatment, the decreased amount of IgA combined with a lowered salivary secretion rate indicate a reduced oral defense with increased risk of oral infections.

## Background

Saliva is produced by the parotid, submandibular, sublingual glands and by a large number of minor glands. The glands contribute in various degrees to the whole saliva, the submandibular/sublingual glands produce 68% of whole saliva in the unstimulated state, the parotid glands 28% and the minor glands 4%. When the glands are stimulated, the parotid glands produce 53% of the saliva, the submandibular/sublingual glands 46% and the minor glands 1%. Unstimulated saliva is viscous, while stimulated saliva is watery [1]. Saliva consists of 99% water and 1% proteins and inorganic salts. Several hundred to thousands of different proteins have been found in whole saliva [2, 3], for example proline-rich proteins, mucins, secretory Immunoglobulin A, lysozyme, lactoferrin and amylase [4]. Saliva has many important functions for oral health [5]. It helps bolus formation of food, aids at mastication and swallowing, and is important for oral clearance (especially stimulated saliva). Saliva covers soft and hard surfaces of the oral cavity and is important for lubrication (especially unstimulated saliva) as well as the protection against pathogenic microbes [6].

An early complication of treatment of cancer of the head and neck region is a reduced salivary secretion rate starting about 2 weeks into radiotherapy [7]. For some patients, a normal stimulated salivary secretion rate can be regained after completed cancer treatment, but about 50% of the patients experience a persistent low stimulated secretion rate (≤ 0.7 mL/min) [8]. The unstimulated salivary secretion rate is mostly very low or even unmeasurable in patients who have undergone treatment of head and neck cancer [9].

A decreased total protein concentration in unstimulated parotid saliva has been previously shown 3–6 months post treatment compared with pretreatment, but it had returned to pretreatment levels at 12–24 months post treatment [10]. No significant changes in total protein concentration in stimulated parotid saliva was detected pretreatment compared with post treatment [10]. However, a higher total protein concentration in unstimulated whole saliva has been reported both pretreatment [11], and 3 months post treatment [12] compared to healthy controls.

The dominating immunoglobulin class in saliva is secretory IgA (SIgA), which is active on mucosal surfaces where it neutralises and eliminates viruses and bacteria. SIgA inhibits microorganisms from adhering to mucosal and dental surfaces [6]. Previous studies have reported diverging results regarding SIgA concentration in saliva in patients with cancer of the head and neck region [10–14].

The most prevalent mucins in saliva are the soluble MUC7 and the gel-forming MUC5B. Both MUC7 and MUC5B are glycoproteins and can interact with oral microbes to facilitate their removal and/or reduce their pathogenicity [15]. Previous studies have shown that patients often report problems with sticky saliva after treatment of cancer of the head and neck region [16, 17]. Our previous study showed that the concentration of MUC5B in stimulated saliva decreases to about 75% of the concentration seen in healthy controls with normal salivary secretion rate [18]. However, there is limited knowledge about changes in salivary composition and mucin structure over time after treatment of cancer of the head and neck region. Such knowledge can contribute to the understanding of patients’ experience of changes in saliva and for assessment of risk of oral diseases.

The aim of the study was therefore to determine over time changes of total protein and IgA as well as mucin type *O*-linked glycans (mostly MUC5B and MUC7) in stimulated whole saliva in relation to dry mouth and sticky saliva in head and neck cancer patients.

## Methods

The present study is part of a larger project approved by the Ethical committee at the University of Gothenburg (Dnr 682-07). Thirty-three patients were recruited at their visit to specialist dentist Bodil Fagerberg-Mohlin before starting cancer treatment. All patients received oral and written information about the project and signed an informed consent form. Their stimulated salivary secretion rate was determined at pretreatment and at 6 months, 1 year, and 2 years post treatment. Clinical status, minor gland secretion rates, stimulated whole salivary secretion rate and microflora on the tongue, buccal mucosa and supragingival plaque and quality of life has been reported previously [8, 17, 19]. In the present study, 29 patients were included. Two patients were excluded because they had no measurable saliva post treatment and two patients because their saliva had not been saved at all time-points.

### Collection of stimulated saliva

Stimulated whole saliva was collected using paraffin wax. The patients chewed on a piece of paraffin until it was soft and then swallowed once. Thereafter the patients chewed on the paraffin wax and saliva was collected in an ice-chilled graded plastic vial during 3 min. The sample was immediately transported to the Department of Oral Microbiology and Immunology where the saliva was transferred to Eppendorf vials, which were centrifuged (Jouan A-14, Frankrike) at 1700×*g* for 7 min. The supernatant was transferred to new Eppendorf vials, which were stored in − 80 °C in a biobank with registration number 607 at the Department of Oral Microbiology and Immunology. The number of samples from each time-point is shown in Table 1.

**Table 1:** Number of samples pretreatment, 6 months, 1 and 2 years post RT

No of patients	Number of samples	No saliva collected
Pretreatment (n = 29)	29	0
6 months post RT (n = 29)	26	3
1 year post post RT (n = 29)	25	4
2 years post post RT (n = 28)	27	2
Total	107	9

In some cases, the patient had no measurable saliva why no saliva could be collected. At 2 years post RT one patient was deceased. For 20 patients, saliva from all four time-points was available for total protein analysis and for 18 patients also for IgA analysis

### Analysis of the total protein concentration

The samples were thawed on ice. The total protein concentration was analysed using the BCA Protein Assay Kit (Pierce, Rockford, IL, USA) with bovine serum albumin (BSA) as a standard. The saliva samples were diluted with distilled water to 1:2 and 1:4. Saliva samples with volumes < 50 µl was diluted 1:10. The saliva samples were analysed in duplicate. All samples from the same patient were analysed in the same plate. After addition of reagents as described by the manufacturer, the plate was incubated for 30 min at 37 °C. The plate was thereafter read at 562 nm in an ELISA reader (Synergy 2, Biotek, Highland park, Winoski).

### Analysis of IgA by sandwich ELISA

Microtiterplates (NUNC, Denmark) were coated with alpha-chain specific anti-human IgA (Sigma, USA) and incubated at 4 °C overnight. After washing with Phosphate buffer (PBS) with 1% Tween-20, saliva samples (thawed on ice) diluted to 1:200 with Bovine serum albumin buffer (BSA-buffer) was added to duplicate wells. Human IgA (Sigma, USA) was used as a standard with a start concentration of 100 ng/mL. BSA buffer was used as a negative control. The plates were incubated in 37 °C during 2 h. After washing, rabbit-anti-human IgA (DAKO, Glostrup, Denmark) was added and the plate was incubated in 37 °C during 2 h. After washing, anti-rabbit-IgG biotin conjugate was added (Sigma-Aldrich). After incubation (2 h, 37 °C) and washing, streptavidin conjugated phosphatase was added and the plates were incubated overnight at 4 °C. After washing, Sigma 104 Phosphatase (Sigma, USA) was added. Finally, the plates were read at 400 nm in the ELISA-reader.

### Determination of mucin type O-linked glycans

Saliva samples from 6 cancer patients were selected. After thawing on ice, 20 µL of each sample was concentrated to 10 µL using SpeedVac (room temperature, 5 min). 20 µL of 7 M urea was added and heated to 95 °C for 15 min. To reduce the proteins, 25 mM dithiothreitol (DTT) was added before heating followed by alkylation using 62.5 mM iodoacetamide. The samples were centrifuged briefly before loading to gradient sodium dodecyl sulfate-agarose/polyacrylamide composite gel electrophoresis (SDS-AgPAGE) as described previously [20]. Periodic acid-Schiff (PAS) was used to stain the carbohydrates on the separated salivary glycoproteins.

### Analysis of released *O*-glycans using LC–MS/MS

36 µL of the salivary sample was dotted to polyvinylidene (PVDF) membrane (Immobilon P membranes, Millipore, Bellerica, MA). *O*-glycans were released from the PVDF membrane strips by reductive β-elimination [20]. For liquid chromatograph–electrospray ionization tandem mass spectrometry (LC–ECI/MS), the released oligosaccharides were injected on to a column (10 cm × 250 µm) packed in-house with 5 µm porous graphite particles (Hypercarb, Thermo-Hypersil, Runcorn, UK). Acetonitrile gradient (Buffer A, 10 mM ammonium bicarbonate; Buffer B, 10 mM ammonium bicarbonate in 80% acetonitrile) was used as eluent. The gradient (0–45% Buffer B) was eluted for 46 min, followed by a wash step with 100% Buffer B, and equilibrated with Buffer A in the next 24 min. A 40 cm × 50 µm i.d. fused silica capillary was used as transfer line.

The samples were analyzed in negative ion mode on an LTQ linear ion trap mass spectrometer (Thermo Electron, San José, CA), with an IonMax standard ESI source equipped with a stainless steel needle kept at—3.5 kV. Compressed air was used as nebulizer gas. The heated capillary was kept at 270 °C, and the capillary voltage was—50 kV. Full scan (*m/z* 380–2000, maximum 100 ms, target value of 30,000) was performed, followed by data-dependent MS^2^ scans (two microscans, maximum 100 ms, target value of 10,000) with normalized collision energy of 35%, isolation window of 2.5 units, activation q = 0.25 and activation time 30 ms. The threshold for MS^2^ was set to 300 counts. Data acquisition and processing were conducted with Xcalibur software (Version 2.0.7). Glycans were identified from their MS/MS spectra by manual annotation validated by available structures stored in Unicarb-DR database (2019-06 version) [21]. The annotated structures were submitted to the UniCarb-DR database and will be included in the next release. The LC/MS ESI data was processed using Progenesis QI (Nonlinear Dynamics, Waters). For comparison of glycan abundance between samples, individual glycan structures were quantified relative to the total content by integration of the extracted ion chromatogram peak area. The area under the curve (AUC) of each structure was normalized to the total AUC and expressed as a percentage. In order to evaluate the alteration of glycan profile before and post treatment, samples collected from different time points from the same patient were analyses individually or as a pooled sample. 40 dominating *O*-glycans of the pooled samples were selected for comparison.

### Assessment of subjective dry mouth and sticky saliva

Data regarding experience of problems with dry mouth and sticky saliva was retrieved from the European Organisation of Research and Treatment of Cancer Quality of life questionnaire (EORTC-QLQ) head and neck module (HN35), which the patients filled in at pretreatment and at 6 months, 1 and 2 years post treatment. In this questionnaire the patient is requested to grade his/her problem with dry mouth and sticky saliva: not at all (1), a little (2), quite a bit (3), very much (4).

### Statistical analysis

Output/min was determined by multiplying concentration with secretion rate. ANOVA was used to analyze differences in total protein concentration and IgA concentration as well as output/min of total protein and IgA between the different time-points and also to determine differences in concentration between those with a secretion rate ≤ 0.7 mL/min and those with a secretion rate > 0.7 mL/min. Regression analysis was used to analyze correlations between dry mouth/sticky saliva and salivary secretion rate/total protein concentration, at 6 months and 1 and 2 years post treatment. ANOVA and Tukey’s multiple comparisons test was used to analyze differences in terminal *O*-glycan modifications i.e. sialylation, sulfation and neutral and in core types between the patients and between time-points. Regression analysis was also used to analyze correlations between terminal *O*-glycan modifications and dry mouth/sticky saliva. *p* values < 0.05 was considered statistically significant.

## Results

The patient group (n = 29) consisted of 20 men and 9 women with a mean age of 59 ± 8 years (range 43–76 years) (Table 2). The mean number of teeth was 25 ± 5. The most common cancer diagnoses were tonsil cancer (n = 18) and tongue base cancer (n = 5). The most common treatment modalities were radiotherapy combined with chemotherapy (n = 23), followed by radiotherapy combined with both chemotherapy and brachytherapy (n = 11) (Table 2).

**Table 2:** Tumor site, gender, age and treatment for the 29 patients

Tumor site	Number (men/women)	Mean age	Chemo-therapy	Surgery therapy	Brachy
Tonsil	18 (10 M/8 W)	58	15	0	7
Tonguebase	5 (4 M/1 W)	62	4	0	4
Oropharyngeal	2 (2 M)	64	2	0	0
Nasopharyngeal	1 (1 M)	43	2	0	0
Tongue	1 (1 M)	74	0	1	1
Tumor colli	2 (2 M)	57	0	2	0

All patients were treated with curative external radiotherapy

### Stimulated salivary secretion

The mean stimulated secretion rate pretreatment was 2.0 ± 0.9 mL/min and only 2 patients had a secretion rate of < 0.7 mL/min. The mean stimulated salivary secretion rate was lowest at 6 months post treatment, 0.6 ± 0.5 mL/min. The majority of the patients (n = 19) had a decreased secretion rate, which was < 0.7 mL/min. At the follow ups at 1 and 2 years post treatment the mean secretion rate had increased slightly, 0.9 ± 0.6 mL/min and 0.9 ± 0.5 mL/min, respectively. Two years post treatment 13 patients (46%) still had a secretion rate of < 0.7 mL/min.

### Total protein concentration and output

**Fig. 1:**
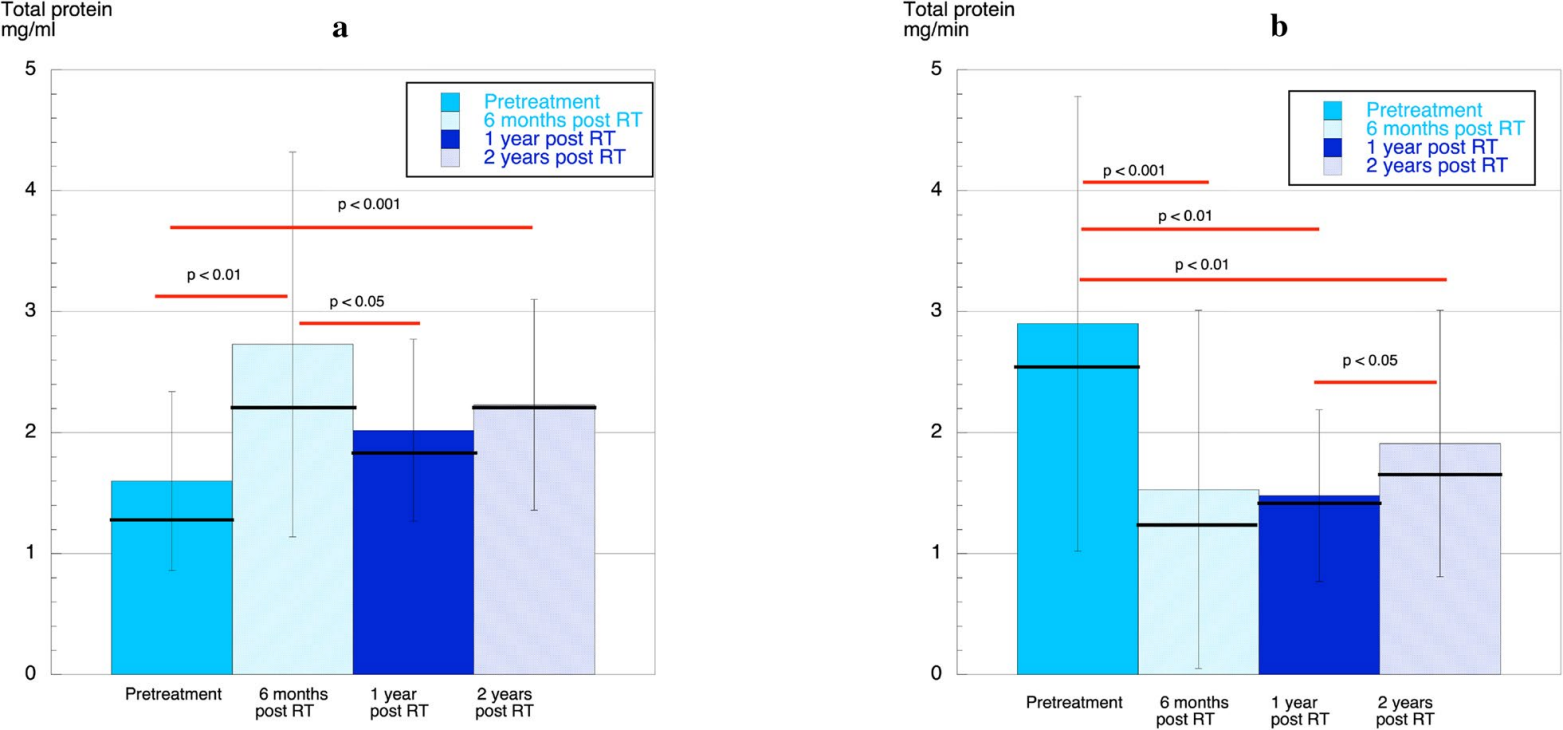
**a** Mean (column), SD (vertical line) and median (horizontal line) total protein concentrations pretreatment and 6 months, 1 year, and, 2 years post treatment. Statistically significant differences between time-points are shown (red line). **b** Mean (column), SD (vertical line) and median (horizontal line) output/min of total protein pretreatment, 6 months, 1 year, and 2 years post treatment. Statistically significant differences between time-points are shown (red line)

The mean total protein concentration was highest at 6 months post treatment (Fig. 1a). At 2 years post treatment, it was still higher compared to pretreatment. For the 20 patients where the total protein concentration was analysed at all four time-points, the lowest mean value was seen at pretreatment 1.6 ± 0.6 mg/mL and the highest at 6 months post treatment 2.8 ± 1.7 mg/mL. At 2 years post treatment, the total protein concentration was still higher compared with pretreatment 2.3 ± 0.8 mg/mL. Figure 1b shows that the total output of proteins (mg/min) was significantly reduced post treatment; at 6 months (1.5 ± 1.5 mg/min, *p* < 0.001), 1 year (1.5 ± 0.3 mg/min, p < 0.01), and 2 years (1.9 ± 1.1 mg/min, *p* < 0.01) compared to pretreatment (2.9 ± 1.9 mg/min).

### IgA concentration and output

**Fig. 2:**
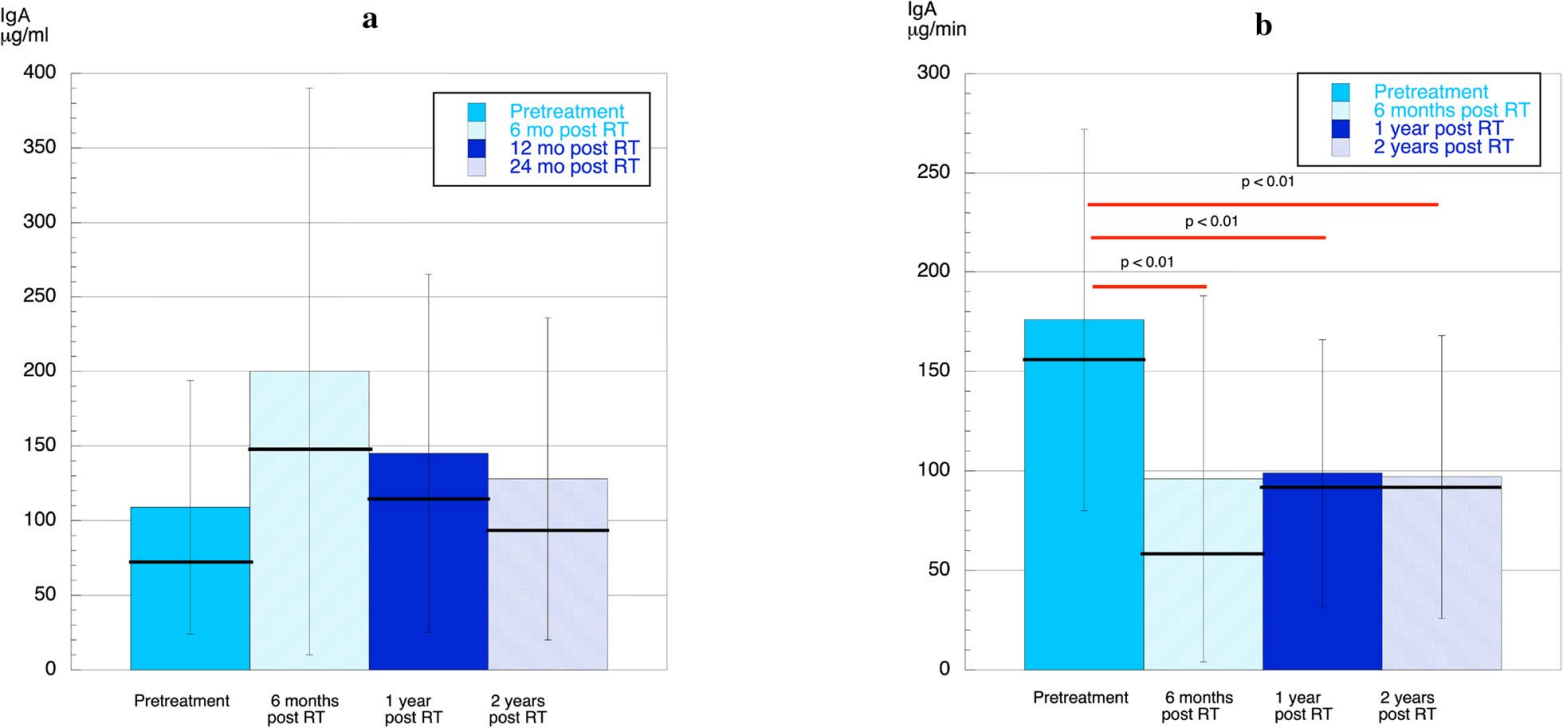
**a** Mean (column), SD (vertical line), and median (horizontal line) concentrations of IgA pretreatment, 6 months**,** 1 year, and 2 years post treatment. No significant differences were detected between time-points. **b** Mean (column) and median (line) output/min of IgA pretreatment and 6 months, 1 year, and 2 years post treatment. Statistically significant differences between time-points are shown (red line)

Due to large variations in IgA concentration between individuals, no significant differences were detected. However, indications were that IgA increased as a response to treatment, but this increase declined over time (Fig. 2a). For the 18 patients where IgA was determined at all four time-points, the highest concentration was seen 6 months post treatment 235 ± 212 µg/mL. At 2 years post treatment, the concentration of IgA was reduced but still higher (124 ± 111 µg/mL compared with pretreatment and 100 ± 53 µg/mL, respectively). However, Fig. 2b shows that the output of IgA (µg/min) was significantly reduced post treatment; at 6 months (*p* < 0.01), 1 year (*p* < 0.01) and 2 years (*p* < 0.01) compared with pretreatment.

### Subjects with a stimulated secretion of < 0.7 mL/min and ≥ 0.7 mL/min

Table 3 shows concentrations and outputs for subjects with hyposalivation (< 0.7 mL/min) and normal salivary secretion (≥ 0.7 mL/min) post treatment. In subjects with hyposalivation, the total protein concentration was significantly higher at 1 and 2 years post treatment, while the IgA concentration showed significantly higher values only at 6 months post treatment. The output/min of total protein was significantly reduced for the hyposalivation group at 6 months and 1 year post treatment, whereas the IgA output was significantly lower only at 1 year post treatment.

**Table 3:** Demographics and salivary secretion rates for patients selected for mucin *O*-glycosylation analysis

Code	Sex	Age	Tumor site	TNM, stage	Treatment	Stimulated salivary secretion rate (ml/min)			
						Pretreat	6 mo	1 year	2 years
1	M	55	Tumor colli	T0N2M0, st IV	S, ERT	2.4	0.5	0.8	1.1
2	M	65	Tonsil	T2N1M0, st II	C, ERT	2.3	0.7	1.9	1.6
3	F	47	Tonsil	T2N0M0, st II	ERT	1.9	0.7	0.6	1.0
4	M	43	Nasopharynx	T3N2M0, st III	C, ERT	2.4	0.7	1.0	1.0
5	F	66	Tonsil	T2N2M0, st IV	C, ERT	1.6	0.3	0.8	0.7
6	M	54	Tonsil	T2N2bM0, st IV	C, ERT	0.9	0.3	0.3	0.5

*S* surgery, *ERT* external radiation therapy, *C* cytostatics

### Mucins

Patients selected for mucin analysis are shown in Table 4. Compared to saliva from a healthy control, all samples showed a lower amount of MUC7 or no detectable amounts of MUC7 on the gels. In most cases, MUC5B from 6 months post treatment showed the highest intensity, while MUC5B before treatment showed low or no signal.

**Table 4:** Problems with dry mouth and sticky saliva for the six patients included in mucin *O*-glycosylation analysis

Code	Dry mouth				Sticky saliva			
	Pretreat	6 mo	1 year	2 years	Pretreat	6 mo	1 year	2 years
1	1	4	4	3	1	2	2	1
2	1	2	2	4	1	1	1	4
3	1	2	2	2	1	3	2	2
4	1	4	3	2	1	2	–	2
5	2	2	2	2	1	1	2	1
6	1	3	3	2	1	3	2	1
Mean	1.2	2.8	2.7	2.5	1.0	2.0	1.6	1.8

1 = not at all, 2 = a little, 3 = quite a bit, 4 = very much

### Glycan analysis

**Fig. 3:**
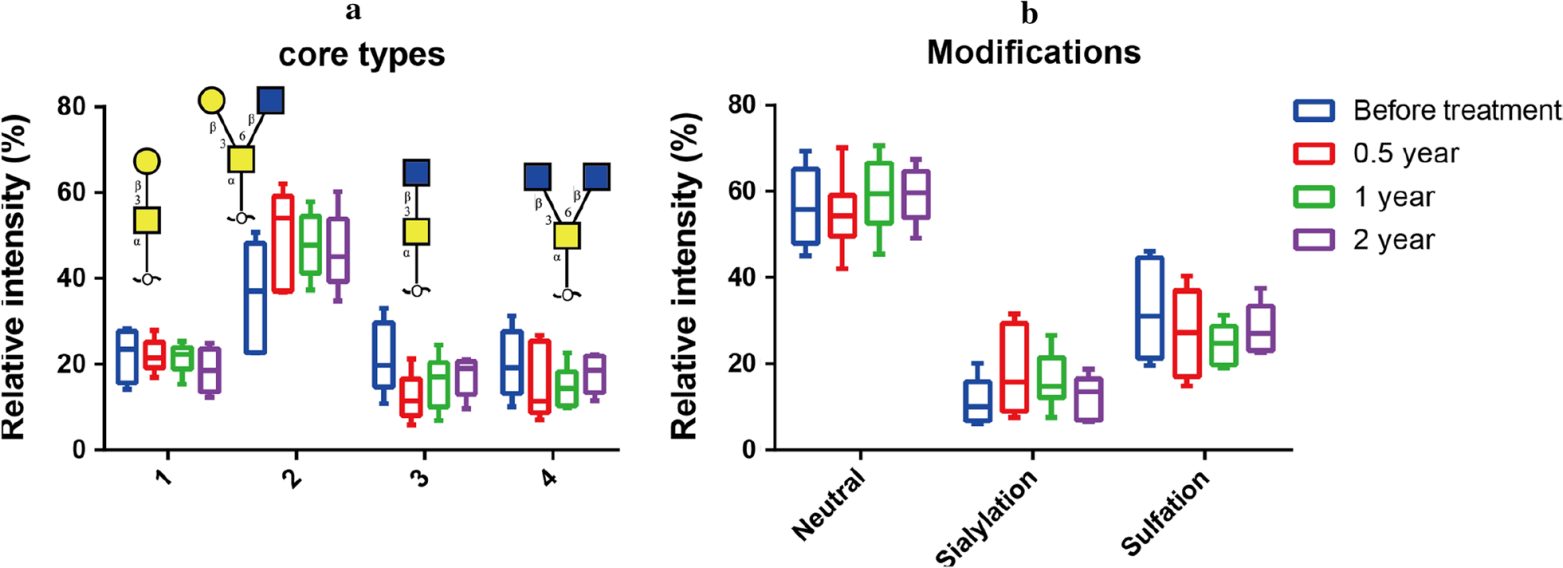
**a** Core types (core 1, 2, 3 and 4) of selected O-glycans pretreatment and at 6 months, 1 year, and 2 years post treatment. Mean and SE are shown. No statistically significant differences were detected. **b** Terminal modification of selected *O*-glycans before treatment and 6 months, 1 year, and 2 years post treatment. Mean and SE are shown. No statistically significant differences were detected

40 dominant glycans of MUC5B were selected for comparison. Five of the 6 patients showed the highest glycan ion intensity (indicative of the highest mucin secretion) in samples collected at 6 months post treatment, the time-point with the highest total protein concentration and the lowest salivary secretion rate. Post treatment, the saliva samples showed a slight increase in the percentage of core 2 structures, at the expense of lower percentage of core structures 1, 3 and 4 (Fig. 3a). There were only minor changes in the mean relative intensity of neutral *O*-glycans at the different time-points (Fig. 3b). Compared with pretreatment, the relative intensity of sialylation was higher at 6 months post treatment, while the relative intensity of sulfation was lower. At 6 months post treatment, the salivary secretion rate was lowest and the patients’ complaints of dry mouth and sticky saliva were highest. At 2 years post treatment, the level of sialylation and sulfation appeared to have returned to pretreatment levels.

### Correlations between experienced dry mouth/sticky saliva and salivary secretion rate/total protein concentration, and between terminal *O*-glycan modifications and dry mouth/sticky saliva

The patients’ problems with dry mouth and sticky saliva are shown in Table 5. As can be seen in Table 6, there were significant positive correlations between the patients’ experience of dry mouth and sticky saliva at 6 months, 1 and 2 years post treatment. There was a tendency to a positive correlation between the measure neutral terminal *O*-glycan modifications and the patients’ experience of sticky saliva (r = 0.764, *p* = 0.08).

**Table 5:** Total protein concentration and IgA concentration as well as output/min of total protein and IgA in subjects with a stimulated salivary secretion rate of < 0.7 mL/min and subjects with a secretion rate of ≥ 0.7 mL/min at 6 months, 1 year and 2 years post RT

	Total protein (mg/mL)	IgA (µg/mL)	Total protein output (mg/min)	IgA output (µg/min)
*6 months post RT*				
< 0.7 mL/min (n = 16)	3.1 ± 1.8	257 ± 215	1.0 ± 0.5	93 ± 109
≥ 0.7 mL/min (n = 13)	2.1 ± 0.9	100 ± 60	2.6 ± 2.1	101 ± 55
	N.S	*p* < 0.05	*p* < 0.01	N.S
*One year post RT*				
< 0.7 mL/min (n = 14)	2.4 ± 0.7	169 ± 142	1.0 ± 0.4	68 ± 46
≥ 0.7 mL/min (n = 15)	1.7 ± 0.7	125 ± 99	2.0 ± 0.6	125 ± 73
	*p* < 0.05	N.S	*p* < 0.001	*p* < 0.05
*Two years post RT*				
< 0.7 mL/min (n = 14)	2.6 ± 1.0	160 ± 123	1.5 ± 1.0	76 ± 64
≥ 0.7 mL/min (n = 15)	2.0 ± 0.7	94 ± 68	2.3 ± 1.1	114 ± 74
	*p* < 0.05	N.S	N.S	N.S

**Table 6:** Correlations between dry mouth/sticky saliva and total protein concentration/salivary secretion rate and between total protein concentration and salivary secretion rate at 6 months, 1 and 2 years post RT

	6 months RT	1 year post RT	2 years post RT
Dry mouth—salivary secretion rate	r = 0.29 (n.s)	r = 0.52 (*p* < 0.05)	r = 0.35 (n.s)
Dry mouth—total protein concentration	r = 0.03 (n.s)	r = 0.19 (n.s)	r = 0.04 (n.s)
Dry mouth—sticky saliva	r = 0.46 (*p* < 0.05)	r = 0.65 (*p* < 0.01)	r = 0.72 (*p* < 0.001)
Sticky saliva—salivary secretion rate	r = 0.12 (n.s)	r = 0.58 (*p* < 0.01)	r = 0.33 (n.s)
Sticky saliva—total protein concentration	r = 0.01 (n.s)	r = 0.05 (n.s)	r = 0.09 (n.s)
Total protein concentration—salivary secretion rate	r = 0.32 (n.s)	r = 0.57 (*p* < 0.01)	r = 0.44 (*p* < 0.05)

## Discussion

The output per min of both total protein and IgA in stimulated whole saliva were decreased post treatment compared with pretreatment. From the gel separation prior to the MS-analysis it was seen that the levels of MUC7 were undetectable or lower compared with saliva from a healthy person both at pretreatment and post treatment. The decrease in protein output per min and in saliva flow in combination with reduced sulfation of MUC5B at 6 months post treatment may contribute to patients’ experience of sticky saliva and oral dryness.

### Methodological considerations

Stimulated whole saliva is easily accessible and consists of saliva produced from all salivary glands. To collect glandular saliva is laborious and time-consuming. Also, besides from collection of saliva the patients in the present study did undergo a clinical oral examination, microbial samples were collected and they filled in several questionnaires. To also collect glandular saliva might have been too time-consuming and tiresome for the patients. Therefor stimulated whole saliva was used for analysis. The mucin analyses were performed on a low number of patients and with different tumor sites. Thus, further studies including a larger number of patients are needed to determine if tumor type and thereby treatment affects mucin *O*-glycosylation post treatment.

### Total protein and saliva secretion rate

To the best of our knowledge, little is known about changes over time in total protein concentration in stimulated whole saliva and even less about output per min of proteins in connection with treatment of cancer of the head and neck region. The present study showed a higher total protein concentration in stimulated whole saliva (1.6 mg/mL; flow rate 2.0 mL/min) for the cancer patients at pretreatment when compared to a previous study including healthy controls (0.9 ± 0.2 mg/mL; flow rate 2.3 mL/min) [18]. The BCA Assay was used in the analysis of total protein both in the present and in our previous study [18]. However, the differences in handling of saliva like centrifugation speed and duration (12,000×*g* for 15 min) in our earlier study, might at least partly explain the lower protein concentration [22]. At the time-points post treatment, the total protein concentration was markedly higher when compared with pretreatment and also compared to healthy controls. However, the output per min was only 62% of the level at pretreatment at 2 years post treatment. In our previous study, the output per min was 63% of the level seen in with controls with normal salivary secretion rate 6 months after completed treatment of head and neck cancer [18]. The reduced salivary secretion rate and output per min of proteins might contribute to the feeling of dry mouth and sticky saliva.

Various studies have showed severe parotid gland hypofunction, exceeding 90%, following irradiation [23]. Moreover, the parotid gland with its high amount of serous type secretory acini is more sensitive to radiation compared with the submandibular and sublingual glands with mostly seromucous and mucous acini [24, 25]. The reduced salivary secretion rate found at post treatment in the current study and saliva with a high protein content might indicate a lower amount of water-rich saliva from the parotid gland, which in turn may also contribute to the feeling of sticky saliva.

### IgA

The concentration of IgA in stimulated saliva was 77.8 ± 107.2 µg/mL (healthy women) and 70.6 ± 54.7 µg/mL (healthy men) [26]. In the present study using the same ELISA technique, we showed a slightly higher concentration of IgA at pretreatment, 110 µg/mL. The concentrations of IgA were higher at all post treatment time-points with large variations and without any significance compared to pretreatment (Fig. 2a). Unstimulated saliva collected from cancer patients pretreatment have shown both a decreased concentration of SIgA [11] and an increased concentration compared with healthy controls [14]. Post treatment, a significantly decreased concentration of IgA in unstimulated saliva was found compared with healthy controls [12]. However, there are no previous studies analyzing IgA concentration or output per min of IgA in stimulated whole saliva in connection with treatment of cancer of the head and neck region. The present study showed an interesting finding that, although the salivary secretion rate has partially recovered 1 year after treatment, the output per min of IgA was 55% of the level seen at pretreatment at 2 years post treatment. This persistent decrease in IgA, might increase the risk of oral infections.

### MUC5B

MUC5B levels in submandibular gland saliva have showed a tendency to be higher among head and neck cancer patients who report no or mild xerostomia 12 months post treatment compared with patients with severe xerostomia who showed almost undetectable levels of MUC5B [27]. Our previous study showed a slightly lower concentration of MUC5B in stimulated whole saliva at 6 months post treatment compared with healthy persons with a normal salivary secretion rates [18]. In the present study a lower abundance of MUC5B was observed at post treatment (data not shown).

Mucins constitute about 16% of the proteins in stimulated saliva [28] and are responsible for hydration and lubrication of mucosal surfaces [29]. Glycans and especially O-linked glycans can inhibit binding of *Candida albicans* to buccal epithelial cells [30]. The 40 most abundant glycans were analysed in the present study. A decrease in sulfation was seen at 6 months post treatment and recovered thereafter. At 6 months post treatment, the salivary secretion rate was lowest and the patients’ complaints of dry mouth and sticky saliva were highest. A reduced level of sulfation of MUC5B has been reported in patients with xerostomia due to Sjögren’s syndrome [31]. MUC5B from the palatal minor glands have a high proportion of sulfation [32]. Hence, it could be speculated that the higher level of sulfation seen at one, and 2 years post treatment is due to an increased contribution of secretion from the palatal glands.

Sialylation has been reported to be most prevalent in MUC5B from the submandibular gland (20%) followed by sublingual gland (11%) and palatal minor glands (6%) [32]. The present study showed that the level of sialylation was increased at 6 months thereafter it decreased. A reduced level of sialylation was previously found in patients who had dry mouth problems and in patients with Sjögren’s syndrome compared with controls with normal salivary secretion rate [33]. The decrease in sialylation observed at 1 and 2 years post treatment may reflect the salivary gland cell damage from radiotherapy resulting in decreased posttranslational processes of glycoproteins, which is an energy consuming process [34]. It is possible that changes in sialylation contribute to the patient’s experience of dry mouth and sticky saliva.

### MUC7

From the gel separation prior to the MS-analysis it was seen that MUC7 was only detected in low abundance and in many cases not at all, which in congruence with the results with a previous study where no MUC7 in unstimulated saliva from head and neck cancer patients pretreatment was found [35]. Conflicting results regarding MUC7 in unstimulated saliva from patients with hyposalivation and/or oral dryness has been reported [33, 36]. Chaudbury et al. [33] reported a trend of increased MUC7 in dry mouth patients, while no differences were reported for patients with Sjögren’s syndrome by Chaudbury et al. [36].

### Factors affecting mucin structure

The present study showed that at post treatment salivary core 2 *O*-glycans were increased, whereas core 1, 3 and 4 *O*-glycans were slightly decreased. The function of these structures in mucin formation and viscosity is yet not known. Sodium ions play an important role in unpacking of secreted MUC5B [37]. The increased osmotic pressure caused by sodium ions attracts water molecules and cause swelling of the mucins [37]. Bicarbonate attracts calcium ions, which are used to pack the mucins within the cells [38], facilitating the unfolding of the mucins when they are secreted [39]. At an acidic pH MUC5B formed condensed structures, while a pH of 7.4 showed MUC5B formed a linear network of polymers [40]. An about fivefold higher mean concentration of calcium, a 25% higher concentration of sodium and a 50% lower concentration of bicarbonate were earlier reported in stimulated whole saliva of patients 6 months post treatment of cancer of the head and neck region compared with controls with normal saliva secretion rate [41]. A significantly lower pH in unstimulated saliva 2 years post treatment of head and neck cancer has also been reported [42]. As a result of radiotherapy, the secretory functional unit of acini in salivary glands degenerate, which cause inflammation in the tissue leading to an apparent increase in salivary sodium and chloride and a decrease in bicarbonate [43]. Accordingly, the changed electrolyte composition together with altered mucin structure may contribute to the patients’ experience of dry mouth and sticky saliva.

When unstimulated saliva was analysed using Cryo-SEM, the mucin networks seen in saliva from healthy persons was not present in saliva from patients who had undergone radiation therapy of the head and neck region [44]. Preliminary results from microscope analysis of stimulated saliva from our head and neck cancer patients show a similar pattern with a lower degree of networks compared with stimulated saliva from healthy persons with normal salivary secretion rate.

### Clinical implications

Persistent low salivary secretion rate is a common complication in patients who have undergone treatment of head and neck cancer and many complain from dry mouth and sticky saliva. There is no clear correlation between the salivary secretion rate and the experience of dry mouth indicating changes in the saliva composition. To analyse proteins in saliva as well as structures of the mucins, which contributes to the lubrication and wetting of the mucosal membranes, is important to get further knowledge about how these factors influence patients’ experience of dry mouth and sticky saliva. Such knowledge can be used in the development of treatment alternatives and more effective products to relieve dry mouth feelings.

## Conclusion

Decreased output per min of protein and decreased salivary secretion rate, as well as reduced sulfation of salivary mucins at 6 months post treatment tended to correlate with the experience of sticky saliva and oral dryness. At 2 years post treatment, the decreased amount of IgA combined with a lowered salivary secretion rate might lead to a reduced defense and an increased risk of oral infections.

## Data Availability

Data are available from the corresponding author on reasonable request.

## Abbreviations

ELISA: Enzyme-linked immunosorbent assay
LC/MS: Liquid chromatograpy/mass spectrometry
SDS-AgPAGE: Sodium dodecyl sulfate-agarose polyacrylamide gel electrophoresis
